# Genomic Insights Into the Admixture History of Mongolic- and Tungusic-Speaking Populations From Southwestern East Asia

**DOI:** 10.3389/fgene.2021.685285

**Published:** 2021-06-22

**Authors:** Jing Chen, Guanglin He, Zheng Ren, Qiyan Wang, Yubo Liu, Hongling Zhang, Meiqing Yang, Han Zhang, Jingyan Ji, Jing Zhao, Jianxin Guo, Kongyang Zhu, Xiaomin Yang, Rui Wang, Hao Ma, Chuan-Chao Wang, Jiang Huang

**Affiliations:** ^1^Department of Forensic Medicine, Guizhou Medical University, Guiyang, China; ^2^State Key Laboratory of Cellular Stress Biology, State Key Laboratory of Marine Environmental Science, Department of Anthropology and Ethnology, Institute of Anthropology, National Institute for Data Science in Health and Medicine, Xiamen University, Xiamen, China; ^3^School of Basic Medical Sciences, Zhejiang University School of Medicine, Hangzhou, China

**Keywords:** population history, genetic structure, genetic admixture, East Asia, population genetics

## Abstract

As a major part of the modern *Trans-*Eurasian or Altaic language family, most of the Mongolic and Tungusic languages were mainly spoken in northern China, Mongolia, and southern Siberia, but some were also found in southern China. Previous genetic surveys only focused on the dissection of genetic structure of northern Altaic-speaking populations; however, the ancestral origin and genomic diversification of Mongolic and Tungusic–speaking populations from southwestern East Asia remain poorly understood because of the paucity of high-density sampling and genome-wide data. Here, we generated genome-wide data at nearly 700,000 single-nucleotide polymorphisms (SNPs) in 26 Mongolians and 55 Manchus collected from Guizhou province in southwestern China. We applied principal component analysis (PCA), ADMIXTURE, *f* statistics, *qpWave/qpAdm* analysis, *qpGraph*, TreeMix, Fst, and ALDER to infer the fine-scale population genetic structure and admixture history. We found significant genetic differentiation between northern and southern Mongolic and Tungusic speakers, as one specific genetic cline of Manchu and Mongolian was identified in Guizhou province. Further results from ADMIXTURE and *f* statistics showed that the studied Guizhou Mongolians and Manchus had a strong genetic affinity with southern East Asians, especially for inland southern East Asians. The *qpAdm*-based estimates of ancestry admixture proportion demonstrated that Guizhou Mongolians and Manchus people could be modeled as the admixtures of one northern ancestry related to northern Tungusic/Mongolic speakers or Yellow River farmers and one southern ancestry associated with Austronesian, Tai-Kadai, and Austroasiatic speakers. The *qpGraph*-based phylogeny and neighbor-joining tree further confirmed that Guizhou Manchus and Mongolians derived approximately half of the ancestry from their northern ancestors and the other half from southern Indigenous East Asians. The estimated admixture time ranged from 600 to 1,000 years ago, which further confirmed the admixture events were mediated *via* the Mongolians Empire expansion during the formation of the Yuan dynasty.

## Introduction

The East Asian continent has abundant ethnolinguistic diversity and profound history of the populations. The Altaic languages, including Mongolic, Tungusic, and Turkic, are widely distributed in northern East Asia, Siberia, and part region of Central Asia. Previous studies from a genetic perspective have mainly demonstrated the northern East Asian affinity of Mongolic and Tungusic–speaking populations based on the genome-wide single-nucleotide polymorphism (SNP) data or sharing IBD fragments (Yunusbayev et al., 2015; Pugach et al., 2016; Jeong et al., 2020; Kilinc et al., 2021). Based on the large-scale sampling of the ancient and present-day populations from Mongolia, Lake Baikal, to Amur River Basin, it is observed that the Mongolians and Tungusic-speaking groups have a higher proportion of genetic component related to the Devil’s Gate people who were early Neolithic hunter–gatherers in northeastern East Asia dating to more than 7.7 thousand years ago (Siska et al., 2017), as well as Mongolians Neolithic people (Jeong et al., 2020; Wang C. C. et al., 2021). The massive migration of Neolithic people between the eastern Mongolians plateau and the Amur River basin had shaped the culture and genetic structure of Bronze Age and Iron Age and even historic pastoralist empires (Xiongnu, Xianbei, Rouran, Khitan, and Uyghur) (Jeong et al., 2020). This identified ancestry component was referred to as the ancient northeast Asian ancestry compared with the ancient components from Ancient Northern Eurasians and also played an important genetic contribution to modern Mongolic and Tungusic speakers. The genetic similarity of Mongolic and Tungusic populations is also shown in a similar pattern of the paternal Y chromosomes (Wei et al., 2017a,b, 2018a; Zhang et al., 2018). The Y-haplogroup C2^∗^, C2a, and C2b have been identified as the founder paternal lineages of the Tungusic population through whole Y-chromosome sequencing (Wei et al., 2018b). Especially, haplogroup C2a-F5484 has contributed largely to both modern Mongolians and Tungusic populations (Liu et al., 2020). Because of the vast geographic distribution, the present-day Mongolian populations in northern East Asia were suggested to have a distinct genetic substructure due to substantial gene flows between northern Eurasian populations in the past as revealed by whole-genome sequencing (Bai et al., 2018; Zhao et al., 2020). Previous genetic surveys mainly focused on the northern Altaic-speaking populations; however, the ancestral origin and genomic diversification of Mongolic and Tungusic–speaking populations from southwestern East Asia remain poorly understood because of the paucity of high-density sampling and genome-wide data.

Guizhou province, located at the eastern end of the Yunnan-Guizhou Plateau, harbors a diverse array of ethnic groups and linguistic backgrounds including the Mongolic and Tungusic languages (Wang Q. et al., 2021). According to local chronicles and folklore, during the Yuan Dynasty, the Mongolian people were recruited to various regions including Guizhou for their southward or westward expeditions^1^, while the settlement of the Tungusic-speaking Manchus in Guizhou was related to the implementation of military plans by the Qing Dynasty. However, the genetic profile of the Manchus and Mongolian speakers in southern China is still very much in its infancy. Here, we generated genome-wide data at nearly 700,000 SNPs in 26 Mongolian and 55 Manchu individuals collected from three populations in Guizhou province and compared with available data of both modern and ancient East Asian individuals to explore their fine-scale population genetic structure.

## Materials and Methods

### Sampling and Genotyping

We collected saliva samples from 26 Mongolians and 55 Manchus in Guizhou province, southwestern China (Supplementary Figure 1). These samples were collected randomly from unrelated participants whose parents and grandparents are Indigenous people and have a non-consanguineous marriage of the same ethnical group for at least three generations. The ethnicities of all participates were used as their self-declaration based on their family migration history and corresponding family records. Our study and sample collection were reviewed and approved by the Medical Ethics Committee of Guizhou Medical University and followed the recommendations provided by the revised Helsinki Declaration of 2000. The participants provided their written informed consent before they were invited to have participated in this study. We used PureLink Genomic DNA Mini Kit (Thermo Fisher Scientific) to extract DNA and measure the concentration *via* the Nanodrop-2000. Infinium^®^ Global Screening Array (GSA, Shenzhen, China) was used to genotype approximately 700,000 SNPs, which covered SNPs from the autosome, Y-chromosome, and merohedral DNA. Raw data in the binary form (bed, bim, and fam) were initial filtered using PLINK 1.9 (Chang et al., 2015) based on our predefined threshold of the genotyping success rate, missing site rates, minor allele frequency, and Hardy–Weinberg equilibrium (–maf 0.01,–hwe 1e-6, mind: 0.01, and geno: 0.01). A final dataset with 6,992,479 SNPs was used to perform the following population genetic analysis.

### Data Merging

We merged our population data of 81 newly genotyped samples with previously published modern and ancient populations from Human Origins (HO) dataset (Patterson et al., 2012) and the 1240K dataset from the David Reich laboratory^2^, and other recently published ancient East Asians populations (Ning et al., 2020; Yang et al., 2020; Wang C. C. et al., 2021). The 1240K dataset harbored higher-density SNP data from ancient populations, especially for the genome-wide ancient data *via* the capture sequence or whole-genome sequence; however, HO dataset not only has all these ancient DNA data but only has more modern population reference data genotyped *via* the Affymetrix HO array, which can provide more representative source population to construct the modern population genetic background. The detailed information of our used reference population data was listed in Supplementary Table 1. We finally generated two combined datasets used in subsequent analysis covering 72,532 in the merged HO dataset and 193,846 SNPs in the merged 1240K dataset, respectively.

### Principal Component Analysis

We carried out the principal component analysis (PCA) using the smartpca package built-in EIGENSOFT (Patterson et al., 2012). We performed PCA based on present-day East Asian populations and then projected the ancient samples onto the basal axis based on the top two components using the lsqproject: YES option, which accounts for samples with substantial missing data. We did not perform any outlier removal iterations (numoutlieriter: 0). We set all other options to the default and assessed the statistical significance with a Tracy–Widom test using the twstats program of EIGENSOFT.

### ADMIXTURE Analysis

To further explore the ancestry composition and genetic similarity of our studied groups with geographically close ancient and present-day populations, we carried out model-based clustering analysis using ADMIXTURE 1.23 (Alexander et al., 2009) by combining the present-day and ancient worldwide populations samples with our 81 individuals. We performed model-based ADMIXTURE analysis based on the unlinked SNP data (–indep-pairwise 200 25 0.4). We ran ADMIXTURE with default fivefold cross-validation (-CV = 5), varying the number of ancestral populations between *K* = 2 and *K* = 20 in 100 bootstraps with different random seeds. We used the unsupervised ADMIXTURE approach, in which allele frequencies for unadmixed ancestral populations are unknown and are computed during the analysis. We used point estimation and terminated the block relaxation algorithm when the objective function delta < 0.0001. We chose the best run according to the highest log-likelihood. We used cross-validation to identify an “optimal” number of clusters. We observed the lowest CV error at *K* = 11.

### Admixture and Outgroup *f*_3_ Statistics

We used the *qp3pop* in ADMIXTOOLS (Patterson et al., 2012) to perform the outgroup *f*_3_ (Reference1, Reference2; Mbuti) to assess the shared genetic drift between reference populations 2 and reference populations 2 since their separation from an African outgroup population of Mbuti using the default parameters. Then, we used the *qp3pop* to perform the admixture-*f*_3_ (Reference1, Reference2; Target populations) to explore the admixture signatures in our studied Guizhou Manchus and Mongolian samples with different Eurasian ancestral source candidates, where a significant negative-*f*_3_ value with |Z-score| larger than three denoted that the targeted population was an admixture between two parental populations.

### *f*_4_ Statistics

We computed *f*_4_ statistics of the form *f*_4_(*X*, *Y*; Test, Outgroup) using the *qpDstat* program in ADMIXTOOLS with default parameters and estimated standard errors using the block jackknife (Patterson et al., 2012). The statistics can show if the population test is symmetrically related to *X* and *Y* or shares an excess of alleles with either of the two.

### *qpAdm* Estimation

We investigated the admixture source numbers, plausible admixture sources, and the corresponding admixture proportions based on *qpWave* and *qpAdm* programs in ADMIXTOOLS (Patterson et al., 2012) using the following outgroups: Mbuti, Papuan, Australian, Mixe, Russia_MA1_HG, Onge, Atayal, Ust_Ishim, Russia_Kostenki14, and China_Tianyuan. Parameter of “allsnps: YES” was used here. We used the spatiotemporally different Yellow River basin farmers as the northern sources and Fujian or Taiwan modern and ancient as the southern sources to perform the two population qpAdm model. To further dissect the admixture proportions from inland or coastal southern East Asians, we additionally included ancient populations from Southeast Asia as the third source to conduct three-way admixture models.

### TreeMix and *qpGraph*

Phylogenetic relationship with migration events among modern East Asians was performed using TreeMix and *qpGraph* to explore admixture models with population splits and gene flow in Manchus and Mongolians. We followed the basic model to reconstruct the deep population genomic history of our targeted populations (Wang C. C. et al., 2021).

### ALDER-Based Admixture Times

Admixture dates from the possible admixture sources for Manchus and Mongolians were estimated using ALDER (Loh et al., 2013). We used geographically different northern and southern East Asians as candidate sources to estimate the admixture time. We used Plink 1.9 (Chang et al., 2015) and our in-house script to calculate the pairwise Fst indexes (Weir and Cockerham, 1984).

### Y-Chromosomal and mtDNA Haplogroup Assignment

There were 26,341 paternal lineages informative SNPs and 4,198 maternal-informative SNPs genotyped *via* the Infinium^®^ GSA. Ancestral or derived statuses of these SNPs were used to identify the terminal haplogroup. We used in-house tools (unpublished software) to assign the Y-chromosomal paternal lineage following the basic regulations reaccommodated *via* the International Society of Genetic Genealogy^3^. We classified the maternal mitochondrial haplogroups used HaploGrep 2 (Weissensteiner, 2016).

## Results

**FIGURE 1:**
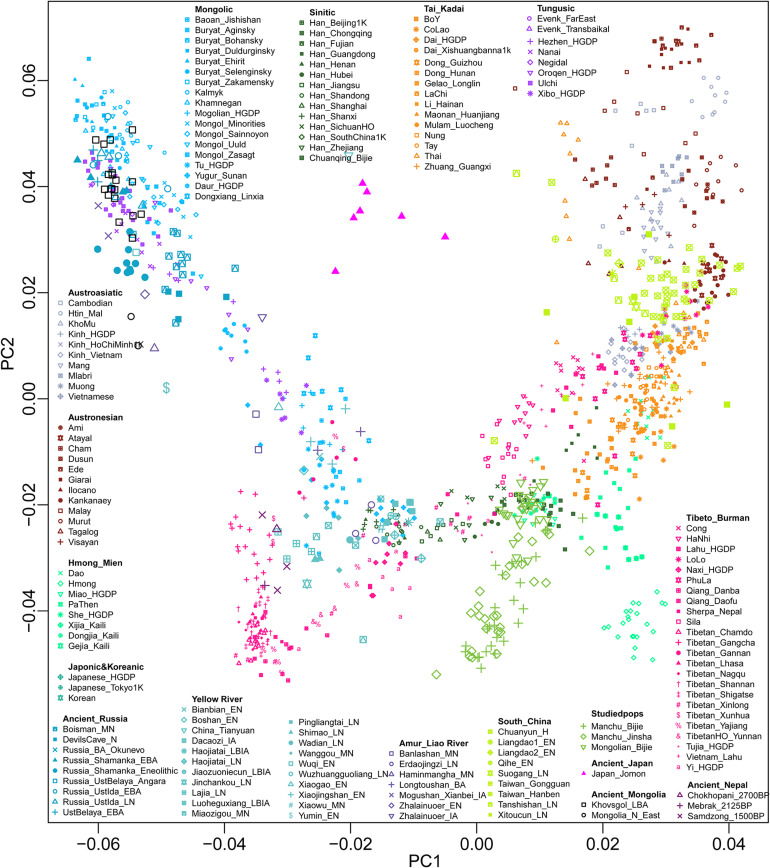
Patterns of genetic relationship among East Asian populations inferred from principal component analysis. Genetic background was constructed based on the genetic variations from modern populations and their top two components. Modern populations were color-coded on the basis of their language family categories. All ancient populations were projected onto it.

We successfully genotyped approximately 700,000 genome-wide SNPs in 26 Mongolians and 55 Manchus in the Guizhou province, China. We then merged our data with worldwide modern and ancient published populations from the HO dataset and 1240K dataset, which included modern populations from Altaic, Sino-Tibetan, Austronesian, Austroasiatic, Hmong-Mien, and Tai-Kadai speakers in East Asia (Wang C. C. et al., 2021), as well as ancient DNA data from Nepal (Jeong et al., 2016), Mongolia (Jeong et al., 2020), Siberia (Lazaridis et al., 2014; Raghavan et al., 2014a,b, 2015; Rasmussen et al., 2014; Mathieson et al., 2015; Damgaard et al., 2018; de Barros Damgaard et al., 2018; Sikora et al., 2019), North and South China (Yang et al., 2017, 2020; Ning et al., 2020; Wang C. C. et al., 2021), and Southeast Asia (Lipson et al., 2018; McColl et al., 2018). To understand the general patterns of relatedness between Guizhou Manchus, Mongolians, and published populations, we first performed PCA to provide a overview pattern of the population structure across East Asia (Figure 1). We observed the following five genetic clusters correlating well with geographic and linguistic categories within East Asia: (I) a northern Altaic cluster consisting of Tungusic and Mongolic–speaking groups in North China, Mongolia, and Siberia; (II) a southern China/Southeast Asia cluster with Austroasiatic, Tai-Kadai, and Austronesian speaking groups; (III) a western Tibetan Plateau cluster being made up of Tibeto-Burman–speaking populations; (IV) a southern inland East Asian Hmong-Mien cluster comprising Hmong, Dao, Gejia, Dongjia, and Xijia; and (VI) a new identified southern Chinese Altaic cluster consisting of Tungusic and Mongolic–speaking groups. Our studied Tungusic and Mongolic–speaking populations from Guizhou province formed a unique genetic cline, which was located at an intermediate position between the western Tibetan Plateau cluster and Hmong-Mien cluster and partially overlapped with previously published Sinitic and Hmong-Mien speaking populations.

**FIGURE 2:**
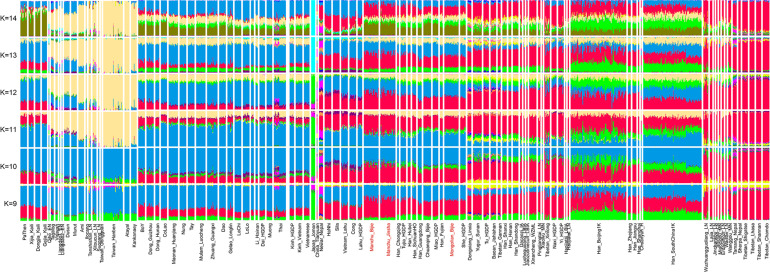
Results of model-based ADMIXTURE clustering analysis. Clustering patterns were visualized with the predefined ancestral sources ranging from 9 to 14 among East Asians (K: 9–14). Here, we can identify late Neolithic to Iron Age Taiwan Hanben dominant ancestry widely distributed in Austronesian speakers, LoChi or Lolo-dominant ancestry maximized in Tai-Kadai–speaking populations, Tibetan-dominant ancestry widely distributed in Tibeto-Burman–speaking populations, and others, all of these ancestries were color-coded by different colors.

In the model-based ADMIXTURE clustering analysis, we used cross-validation to identify an “optimal” number of clusters. We observed the lowest CV error at *K* = 11. At *K* = 11, we observed three ancestral components in our studied Guizhou Manchus and Mongolian samples (Figure 2). One of these components is enriched in the ancient Nepalese and also found at the highest proportions in Tibetans, with the second component with maximum representation in the Tai-Kadai- and Austroasiatic-speaking populations. The remaining ancestry component in our studied populations was maximized in Austronesian speakers and also enriched in ancient samples from southeast China including Fujian and Taiwan. In general, we found our Manchus and Mongolians are genetically similar to the Hmong-Mien–speaking populations and Han Chinese in South China.

**FIGURE 3:**
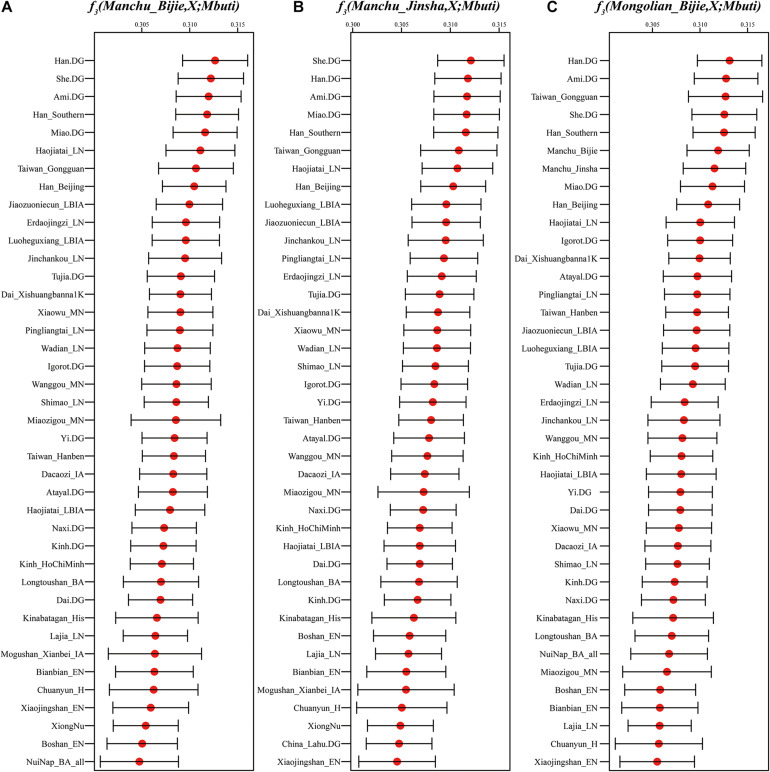
Shared genetic drift estimated *via* admixture-*f*_3_ statistics in different testing forms based on the merged 1240K dataset focused on Bijie Manchus **(A)**, Jinsha Manchus **(B)**, and Bijie Mongolians **(C)**. Bar denotes the three times of error bar.

To formally test the genetic affinity observed in PCA and ADMIXTURE and find the potential ancestral sources for Guizhou Manchus and Mongolians, we measured allele sharing and admixture signals *via* outgroup *f*_3_ and admixture-*f*_3_ statistics. Specifically, in the outgroup *f*_3_ statistics of the form *f*_3_(*X*, Guizhou Manchus/Mongolians; Mbuti), Guizhou Manchus shared more alleles with Han Chinese, She, Ami, and Miao. When *X* represented ancient individuals, Guizhou Manchus was found to share more alleles with Neolithic-Iron Age Yellow River farming populations including Haojiatai, followed by Jiaozuoniecun and Luoheguxiang ancients. Guizhou Mongolians shared more alleles with Han Chinese, Ami, ancient Gongguan samples from Taiwan, She, and Miao (Figure 3 and Supplementary Table 2A). Besides, we used admixture-*f*_3_ statistics of the form *f*_3_(*X*, *Y*; Guizhou Manchus/Mongolians) to model possible admixtures, where *X* and *Y* were East Asian populations that might be the source candidates for modeling the admixture in Guizhou Manchus or Mongolians when getting negative *Z* scores. However, we observed only one significant signal of admixture (*Z* < −3) in the Mongolian_Bijie when using Tibetan as the northern East Asian source and Austronesian-speaking Igorot people as the southern East Asian source (Supplementary Tables 2B–D). This suggests that the allele frequencies of Mongolian_Bijie are intermediate between those of a northern group related to Tibetans and a southern group related to the Austronesian-speaking people. We also calculated pairwise Fst genetic distances among these populations (Supplementary Table 3), and the patterns observed here were consistent with the *f*_3_-based results.

We then performed *f*_4_ statistics to explore genetic substructure between studied groups and other modern/ancient East Asians in the form *f*_4_ (*study group 1*, *study group 2*; *East Asians*, *Mbuti*). We observed significant negative *f*_4_ values in *f*_4_ (*Manchu_Jinsha*, *Mongolian_Bijie*; *East Asians*, *Mbuti*) (Supplementary Table 4A) when we used ancient Hanben samples from Taiwan, Atayal, and early Neolithic Liangdao1 people in the position of “East Asians,” showing that Bijie Mongolians shared the most derived alleles with ancient or modern southern East Asians compared with Jinsha Manchus. We have not observed significant *f*_4_ values in *f*_4_ (*Manchu_Jinsha*, *Manchu_Bijie*; *East Asians*, *Mbuti*) (Supplementary Table 4B), suggesting Manchus from Jinsha and Bijie form a clade with a closer genetic relationship compared with other East Asians. We observed suggestive evidence that Bijie Mongolians may obtain additional gene flow from southern East Asians compared with Bijie Manchus by finding of marginal negative *Z* scores of *f*_4_ (*Manchu_Bijie*, *Mongolian_Bijie*; *East Asians*, *Mbuti*) (Supplementary Table 4C).

We found that Guizhou Manchu and Mongolian people harbored more northern Mongolic and Tungusic–related ancestry compared with Guizhou indigenous populations by the observation of significant positive values in *f*_4_ (*Guizhou Manchus/Mongolians*, *Guizhou indigenous populations*; *northern Mongolians/Tungusic populations*, *Mbuti*) (Supplementary Table 5). Further evidence demonstrated that studied populations harbored more southern East Asian–related ancestry compared to ancient northern East Asians *via* the significant negative *f*_4_ statistics in the form *f*_4_ (*ancient Yellow River millet farmer*, *Guizhou Manchus/Mongolians*; *southern East Asians*, *Mbuti*) (Supplementary Table 6). Compared with ancient populations in southeast China including Fujian and Taiwan, we observed that Guizhou Manchus and Mongolians shared more alleles with northern East Asians *via* significant negative *f*_4_ values in from of *f*_4_ (*Taiwan_Hanben/Xitoucun_LN/Tanshishan_LN*, *Guizhou Manchus/Mongolians*; *northern East Asians*, *Mbuti*) (Supplementary Table 7). Similarly, when compared with present-day southern Sinitic, Austronesian, Tai-Kadai, Hmong-Mien–speaking populations from southern China and the Islands of Southeast Asia, Guizhou Manchus and Mongolians have excess allele sharing with northern East Asians (Supplementary Table 8).

Considering the observed excess allele sharing and possible sources for our studied Manchus and Mongolians people, we applied *qpWave* and *qpAdm* methods to model their ancestry. We used all available ancient northern populations (Bianbian, Boshan, Xiaogao, Xiaowu, Luoheguxiang, Dacaozi_IA, Longtoushan_BA, Shimao_LN, Miaozigou_MN, and Yumin_EN) as the northern sources and Iron Age Hanben samples from Taiwan as the southern sources to estimate the admixture proportions. The Southern East Asian Hanben-like ancestry proportion spanned from 16.5 to 35.7% when using Yellow River farmers as the northern source, whereas the proportion reached 56.7% when using Yumin_EN (hunter–gatherers in Inner Mongolia) (Supplementary Table 9A). To explore if there was any genetic influence from inland southern East Asians related to Austroasiatic speakers, we conducted three-way admixture models by adding ancient Southeast Asians as a third source. The best-fitted three-way admixture proximal models for Manchus and Mongolians are as deriving ancestry from northern ancient Yellow River farming populations, Austronesian-related ancient Southern East Asians (Taiwan_Hanben/Gongguan, Xitoucun), and Austroasiatic-related ancient Southeast Asians (GuaCha_LN, MaiDaDieu_LN, ManBac_LN, NamTun_LN, PhaFaen_Hoabinhian, and TamHang_BA) (Supplementary Table 9B).

**FIGURE 4:**
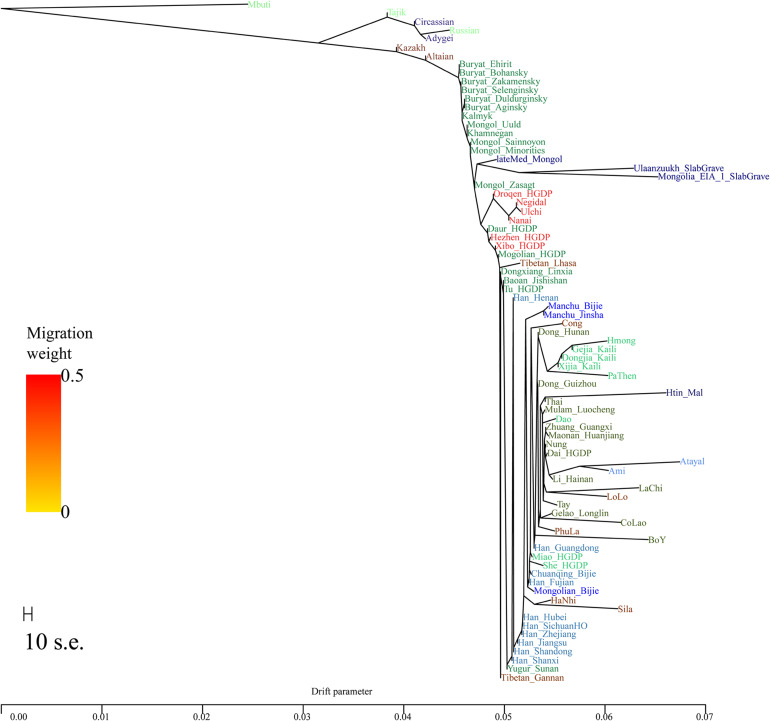
Phylogenetic relationship among northern Altaic, central Sino-Tibetan and southern Austronesian, Austroasiatic, Hmong-Mien, and Tai-Kadai speakers. Mbuti population from central Africa were set as the root. Different populations were marked according to their linguistic affinity.

In the TreeMix analysis (Figure 4), we observed Mongolian-speaking groups in southern Siberia and Tungusic-speaking groups in the Amur River basin cluster together as the northern branch, while the Austronesian, Austroasiatic, Hmong-Mien, and Tai-Kadai speakers from southern China cluster together forming the southern branch. Our studied Mongolians and Manchus groups, Tibeto-Burman and Sinitic populations were located at an intermediate position between the northern and southern branches. Specifically, the two Guizhou Manchus groups in this study clustered together first and then clustered with the Guizhou Mongolians group at an intermediate position between the Sinitic and Hmong-Mien–speaking populations. The clustering pattern was consistent with the patterns observed in the aforementioned PCA, ADMIXTURE, and *f* statistics–based analysis that Guizhou Manchus and Mongolians had experienced genetic influence from surrounding southern Indigenous populations since their separation from northern ancestors and migrated to Guizhou.

**FIGURE 5:**
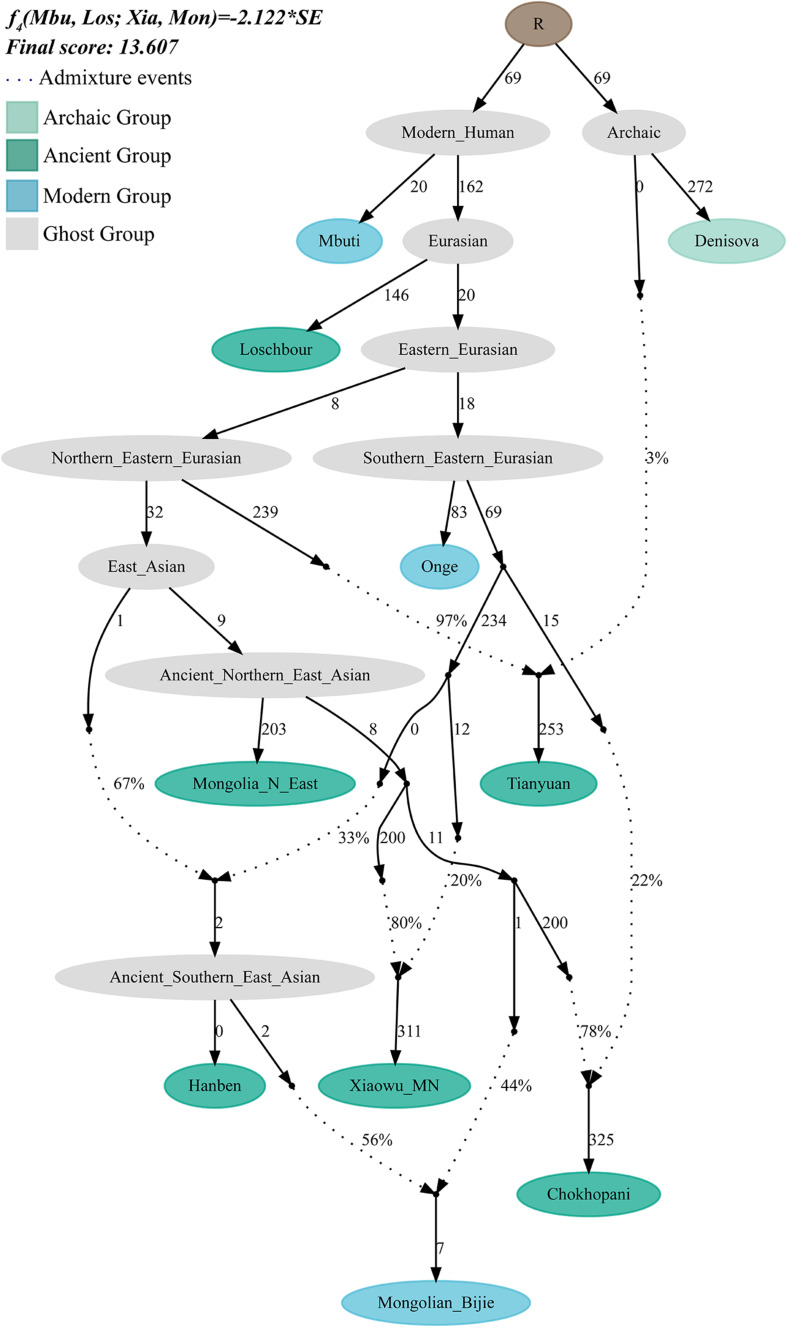
The suggested admixture model of southern Mongolian people *via qpGraph*. The merged 1240K dataset was used. Dotted line denotes the admixture events, and their corresponding admixture proportions also marked. One hundred times of genetic drift (*f*_2_ values) were denoted. Ancient populations, modern targeted, and ghost populations were color-coded.

We further used *qpGraph* to reconstruct the deep evolutionary history of the Mongolians group in Guizhou. We used two ancient Neolithic samples from the Mongolians Plateau as the northern source and used the samples from the middle Neolithic Xiaowu site as a representative of the ancient Yellow River millet farmers. We used Iron Age Hanben samples from Taiwan as the southern source. The reconstructed phylogeny showed that the genetic contribution of the ancient northern East Asians to the Bijie Mongolians is 44%, whereas the proportion from the southern East Asians is approximately 56% (Figure 5).

**FIGURE 6:**
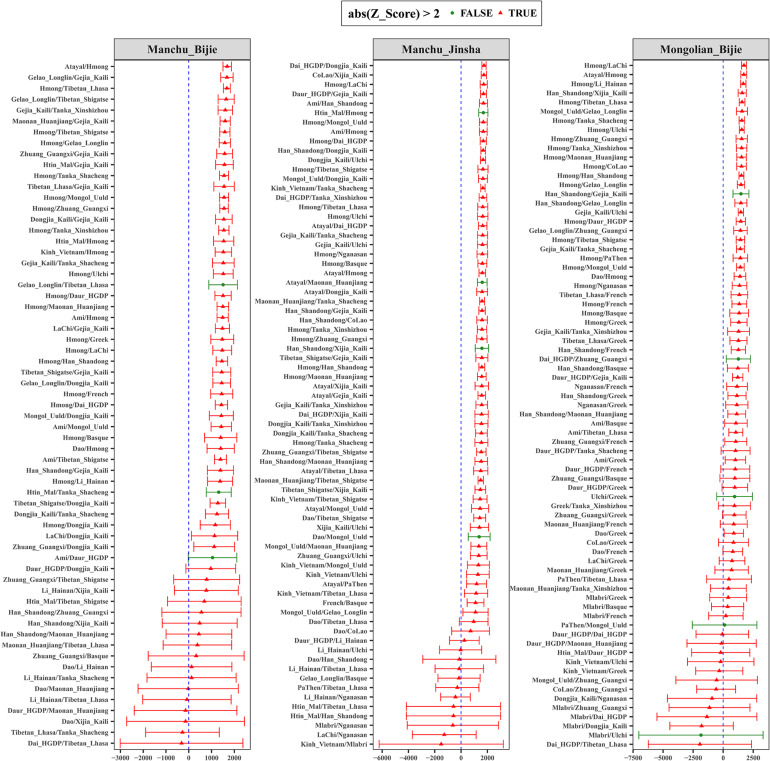
ALDER-based admixture time with different ancestral sources. The length of 29 years of one generation was used.

We next used ALDER software to estimate when the admixture occurred. We tried different modern populations from the north and south of East Asia as possible ancestral groups. We observed that most of the average time that admixture occurred is around 1,000 AD, which is concordant with the historically documented expansion of the Mongol Empire and the establishment of the Yuan Dynasty (Figure 6).

We successfully obtained 62 uniparental Y-chromosome lineages and 81 mtDNA lineages. Among 55 studied Manchus samples, we identified 37 maternal lineages with terminal lineage frequencies ranging from 0.0182 to 0.0727 (B4g:4, F1a:4, F1a1:4), and B4, B5a, F, D4, and M7 were the dominant maternal lineages. We obtained 14 terminal paternal lineages among 43 males with frequencies ranging from 0.0233 to 0.3953 (O1b1a2a1-F1759/F2064/CTS5847/CTS8414/Z24393/F3314/F3323/CTS118 90/F3478′: 17). We also identified some Manchus samples with paternal haplogroup C2c1b7∼-Z45293′. For the studied Mongolians, we identified 23 different maternal lineages with frequencies ranging from 0.0384 to 0.0769 (M7b1a1e1: 3), with A5b1, B5a1c1, and M7b1a1 identified at least twice among the Mongolian samples. The high-frequency paternal lineages of our Mongolian samples are O1b1a1a1a1a2a1-Z24050′ (11) and O1a1a2a1-Z23266 (6) (Supplementary Table 10). We also made population compassion among paternal and maternal lineages from ethnically and geographically Guizhou populations; population clustering patterns showed that Mongolic and Tungusic–speaking populations had a close relationship with geographically close populations, suggesting extensive population admixture occurred among them (Supplementary Figures 2–3).

## Discussion

Strong associations between population genetic structure and linguistic similarity were subsequently evidenced among Afroasiatic, Nilo-Saharan, Niger-Congo, and Khoisan language families in Africa (Martin et al., 2018; Patin and Quintana-Murci, 2018; Gurdasani et al., 2019), as well as language families in Asia (Chen et al., 2019; He et al., 2020a,b,c). Recent genome-wide modern and ancient DNA data have demonstrated that obvious population stratifications existed in East Asia with four regional dominant ancestries. The 7,000-year-old eastern Mongolians Neolithic people–related ancestry was widely distributed in modern Tungusic and Mongolic speakers in northern and northeastern China, Mongolia, and southern Siberia (Ning et al., 2020; Wang C. C. et al., 2021). The Tibetan-related ancestry, which was represented by Neolithic Upper and Middle Yellow River farmers, was widely distributed in modern Tibetan-Burman–speaking populations and also a dominant component in Sinitic speakers (Jeong et al., 2016; Massilani et al., 2020; Zhang and Fu, 2020; Wang C. C. et al., 2021). For southern China and Southeast Asia, one ancestry component was widely distributed in Hmong-Mien–speaking populations mainly collected from Guizhou province and Vietnam (Lipson et al., 2018; McColl et al., 2018; Yang et al., 2020; Wang C. C. et al., 2021). The other southern ancestry was dominated in Austronesian-speaking populations (Lipson et al., 2018; McColl et al., 2018; Yang et al., 2020; Wang C. C. et al., 2021), also dominant in Tai-Kadai–speaking Li in Hainan island (He et al., 2020b). However, some exceptions were also identified in China, which may be caused by large-scale population movements and genetic admixture events in the recent and prehistoric time, for example, the East–West admixture along the Silk Road (Yao et al., 2021), and some western Eurasian ancestry was also identified in Iron Age Xinjiang people (Ning et al., 2019). Ancient genome data in East Asia also have illuminated three main Neolithic population expansions that have participated in the formation of modern observed distributed patterns of genetic structure and language families (Wang C. C. et al., 2021). Holocene population movements from the Amur River basin and eastern Mongolia Plateau were associated with the formation of the genetic structure of Mongolic and Tungusic–speaking populations. Similarly, population expansion from the Yellow River basin and the Yangtze River basin, respectively, contributed to the formation of Sino-Tibetan speakers (Wang et al., 2020) and other southern East Asians, as well as the Southeast Asians (Larena et al., 2021; Wang C. C. et al., 2021).

Here, we presented the fine-scale genetic structure of Mongolic and Tungusic–speaking populations (Mongolians and Manchus) in Guizhou and reconstructed their demographic history. We observed significant genetic differences between southern Mongolic and Tungusic speakers from Guizhou and their counterparts from northern East Asia (North China, Mongolia, and southern Siberia). We observed two different genetic clines among all Mongolic and Tungusic–speaking populations in the PCA plots, and Guizhou populations have deviated to the southern East Asian clusters comprising Austronesian, Austroasiatic, and Tai-Kadai populations, as well as close to Hmong-Mien clines. However, northern Mongolic and Tungusic speakers formed another genetic cluster that was located far away from the southern ones. We identified different ancestry components in northern and southern populations in the model-based ADMIXTURE results with the studied Guizhou populations sharing similar genetic profiles with southern East Asians. We observed suggestive evidence in *f*_3_ statistics that Guizhou Manchus and Mongolians derived ancestry from both northern and southern East Asia. But for the northern Mongolic and Tungusic–speaking populations, we can find significant admixture signatures with one source from East Asians and the other from western Eurasians or northern Siberians. The genetic distance-related indexes (Fst and outgroup *f*_3_ statistics) consistently supported the studied Guizhou populations having a strong southern East Asian affinity, but northern Mongolic and Tungusic speakers showing a clear northern East Asian affinity. We observed the Y-chromosome and mtDNA haplogroups in Guizhou Manchus and Mongolians are the lineages that are frequent in southern China, showing a different genetic profile from that in northern Mongolic and Tungusic speakers. Recent genetic studies focused on northern Mongolian and Manchu populations found that paternal lineages of C2a and C2b were widely distributed in these populations, which is rarely found in our focused Guizhou Manchus and Mongolians.

Furthermore, we also identified the genetic differences between studied Manchus and Mongolians with southern East Asians. Our studied Manchus and Mongolians did not group together with geographically close Guizhou populations, such as Guizhou Han, Chuanqing, Gejia, Gongjia, and Xijia. Compared with southern East Asians, Guizhou Manchus and Mongolians shared excess alleles with northern Mongolic/Tungusic–speaking populations, as shown in significant negative *f*_4_ values in *f*_4_ (southern East Asians, studied Guizhou populations; northern East Asians, Mbuti). The *qpGraph*-based phylogeny with admixture events further showed a large proportion of the ancestry of Guizhou Mongolians derived from Yellow River farmers, who were genetically close to Mongolians Neolithic populations. The ALDER-based estimates of admixture times ranged from 500 to 1,500 years ago, which was consistent with the time of Mongolians Empire expansion and the formation of the Yuan dynasty. The excess affinity of Guizhou Manchus and Mongolians with northern populations, when compared with Guizhou Indigenous groups, highlights the role of the southern expansion of northern Mongolians.

Previous genetic, linguistic, and archeological documents from Guizhou and other southwestern China showed that Southwestern East Asia had the highest diversity in genetics, language, and culture. Thus, these complex mixture natures promote the admixture process between southward migrated Manchus and Mongolians and local populations. These strong genetic affinities also supported *via* genome-wide data or traditional genetic markers from southwestern populations (Chen et al., 2018a,b; He et al., 2019, 2020b,c, 2021). However, both of our ALDER-based admixture dates and historically documented migration history of Mongolians in the Yuan Dynasty and Manchus in the Qing Dynasty showed the plausible admixture events that occurred recently. Cultural anthropologies also showed these migrated populations had their specific lifestyles, language, and other customs. Besides, the relatively isolated resediment environments further confirmed some extent genetic isolation between Mongolians, Manchus, and other geographically close populations. It is interesting to identify the genetic affinity between our studied population and Hmong-Mien–speaking populations; one possible reason is that Hmong-Mien–speaking populations are the dominant Indigenous populations directly descended from the ancient Neolithic rice farmer in the middle Yangtze River and may be the direct descendants of the Daxi culture, which provided the typical ancestral component for modern southwestern populations and is also the best surrogate source populations for our studied populations. Indeed, these admixture signatures can be identified *via* admixture-*f*_3_ statistics. Further work should be focused on the whole-genome sequencing data of more Hmong-Mien, southern Mongolic and Tungusic, and ancient DNA data from the higher time-transect to comprehensively characterize the fine-scale demographic history of southern Manchus and Mongolians and other Southeastern Asians.

## Conclusion

We presented the first batch of genome-wide data focusing on the southern Mongolians and Manchus from Guizhou province. We used comprehensive population genetic analyses of PCA, ADMIXTURE, *qpAdm*, *qpWave*, *qpGraph*, and ALDER to explore the complex genetic history and dynamic admixture process of southwestern Chinese populations. We identified one unique genetic cline forming by our studied Mongolians and Manchus samples, which was close to the southern Hmong-Mien cline and Austronesian/Austroasiatic cline but distinct with northern Mongolic and Tungusic cline, suggesting southern Mongolians and Manchus people have experienced a differentiated demographic history since their separation from northern groups. Furthermore, allele-shared–based analysis from *f* statistics revealed that significant admixture occurred in Guizhou Manchus and Mongolians; results from admixture models demonstrated that Guizhou Mongolic and Manchus people harbored both northern ancestry and also additional gene fluxes from southern East Asians. Finally, estimates of ALDER-based admixture times from historic times demonstrated that the presented-day genetic structure observed here was caused by the massive southward migration of Mongolians empire expansion, which is consistent with the historically documented migration events.

## Data Availability Statement

The datasets presented in this study can be found in online repositories. The names of the repository/repositories and accession number(s) can be found below: https://zenodo.org/record/4632918, doi: 10.5281/zenodo.463291.

## Ethics Statement

The studies involving human participants were reviewed and approved by the Medical Ethics Committee of Guizhou Medical University and Xiamen University (Approval Number: XDYX2019009). The patients/participants provided their written informed consent to participate in this study.

## Author Contributions

C-CW and JH designed this study. JC, GH, and C-CW wrote the manuscript. QW, ZR, HLZ, YL, MY, JJ, and JH collected the samples. QW, ZR, HZ, JJ, YL, MY, JC, and JH conducted the experiment. JZ, GH, JG, XY, JC, KZ, RW, HM, and C-CW analyzed the data. All authors reviewed the manuscript.

## Conflict of Interest

The authors declare that the research was conducted in the absence of any commercial or financial relationships that could be construed as a potential conflict of interest.

## Footnotes

1 https://en.wikipedia.org/wiki/Yuan_dynasty

2 https://reich.hms.harvard.edu/downloadablegenotypes-present-day-and-ancient-dna-data-compiled-published-papers

3 https://isogg.org/

## Supplementary Material

Supplementary Figure 1 Geographical location of newly collected samples.

Supplementary Figure 2 Principal component analyses (PCA) among 18 ethnic groups in Guizhou based on the Y-chromosome haplogroup.

Supplementary Figure 3 Principal component analyses (PCA) among 18 ethnic groups in Guizhou based on mitochondrial DNA haplogroup.
