# Supplementary figures and images for: Genomic Insights Into the Admixture History of Mongolic- and Tungusic-Speaking Populations From Southwestern East Asia
Source: Front Genet. 2021 Jun 22;12:685285. doi: 10.3389/fgene.2021.685285 (PMC8258170; doi:10.3389/fgene.2021.685285)

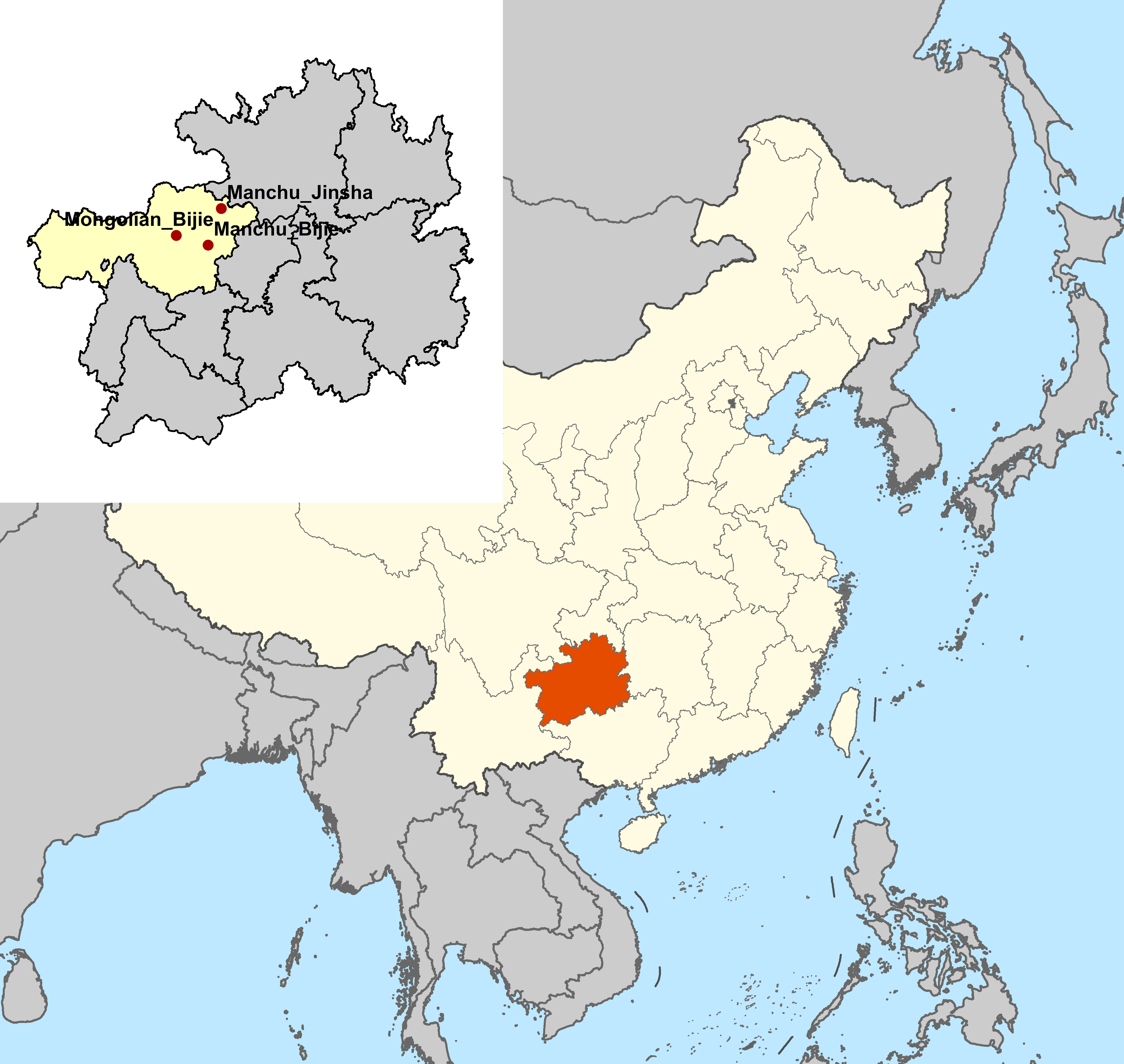

Supplement: Supplementary Figure 1 — Geographical location of newly collected samples. [file Image_1.TIF]

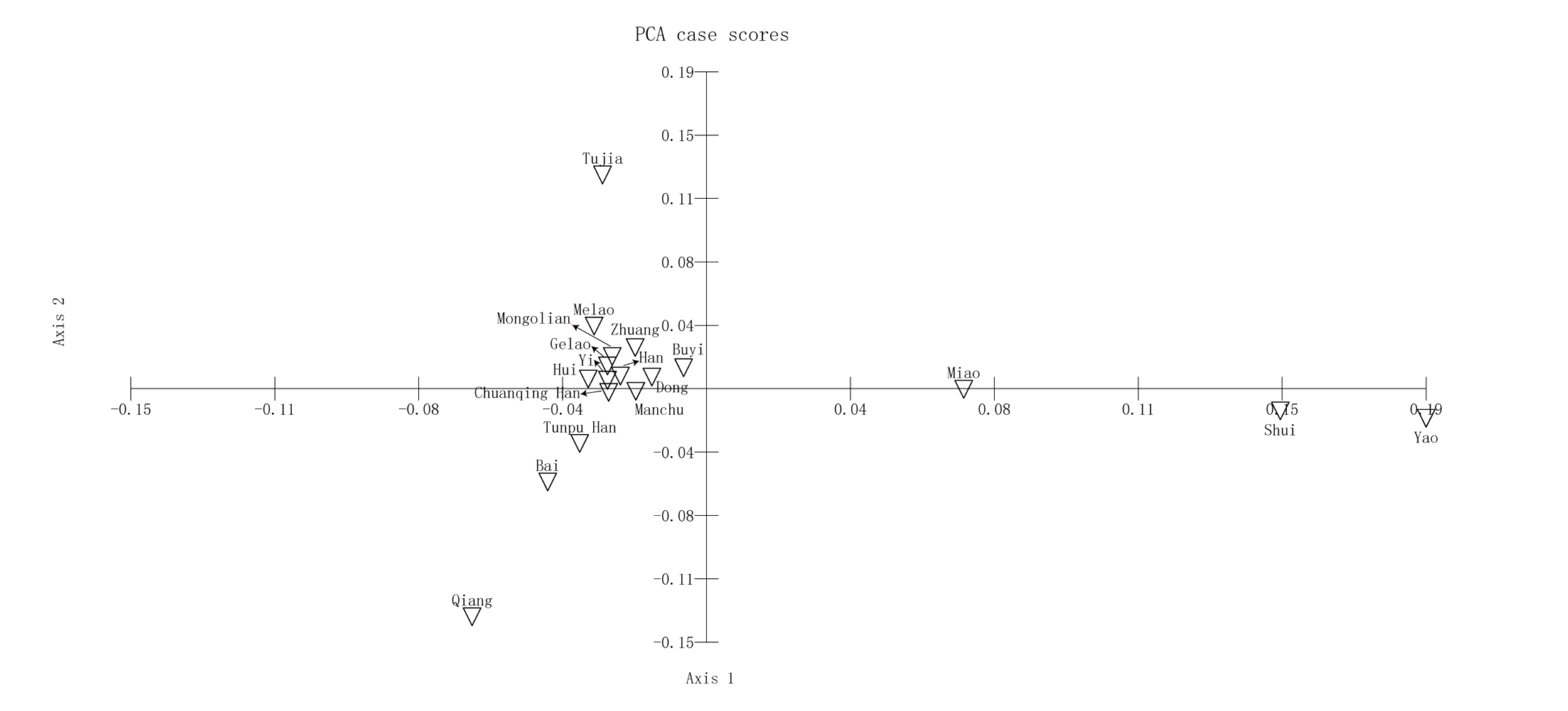

Supplement: Supplementary Figure 2 — Principal component analyses (PCA) among 18 ethnic groups in Guizhou based on the Y-chromosome haplogroup. [file Image_2.TIF]

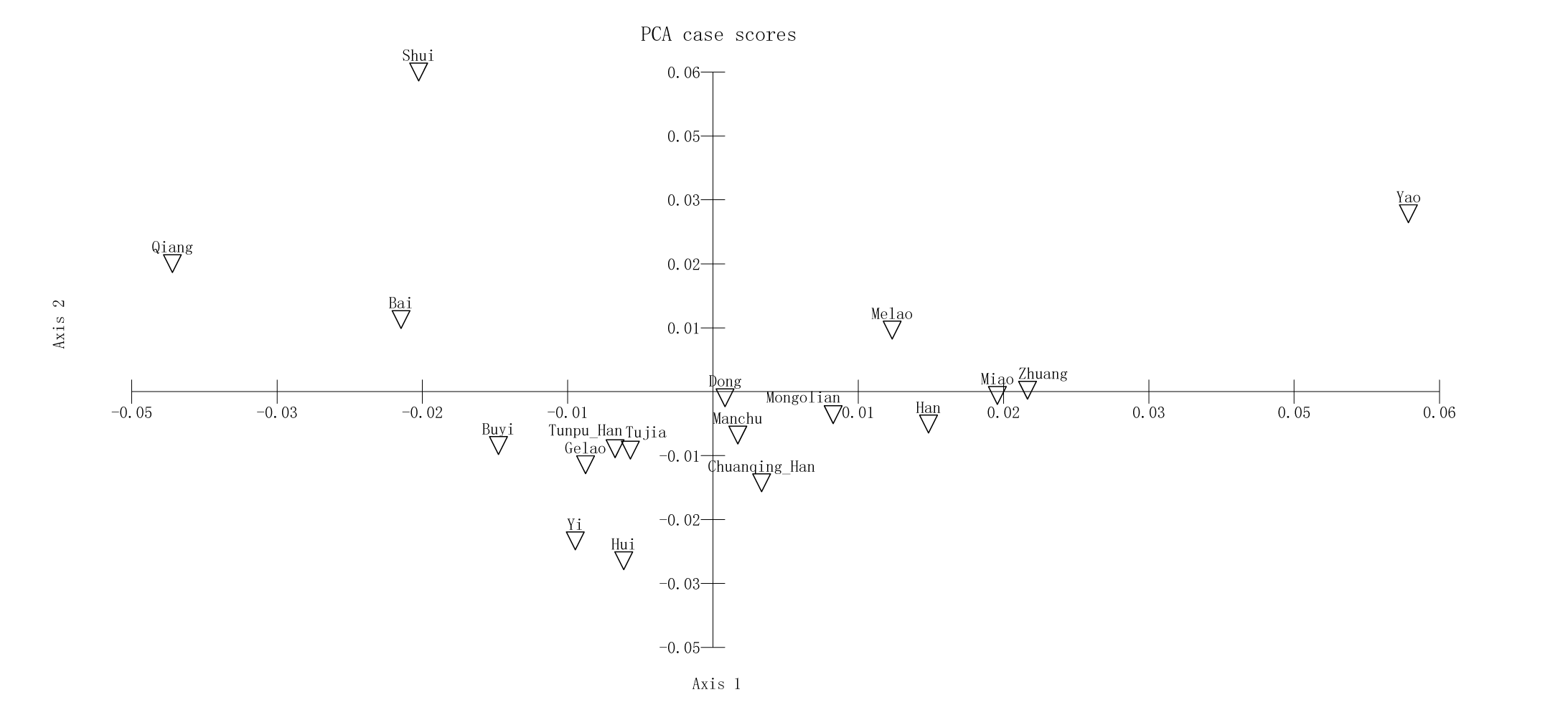

Supplement: Supplementary Figure 3 — Principal component analyses (PCA) among 18 ethnic groups in Guizhou based on mitochondrial DNA haplogroup. [file Image_3.TIF]
